# Development and validation of an interpretable machine learning model for non-invasive screening of precancerous gastric lesions using symptom and lifestyle data: a multicentre cohort study

**DOI:** 10.1016/j.eclinm.2026.103756

**Published:** 2026-01-17

**Authors:** Lan Wang, Kaiqiang Tang, Peng Zhang, Jiasheng Liu, Bowen Wu, Jun Chen, Yan Li, Shiyu Du, Yan Wang, Shao Li

**Affiliations:** a Institute for TCM-X, MOE Key Laboratory of Bioinformatics, Bioinformatics Division, BNRIST, Department of Automation, Tsinghua University, Beijing, China; b School of Robotics and Automation, Nanjing University, Nanjing, China; c Institute of Population Medicine, School of Public Health, Fujian Medical University, Fuzhou, China; d Department of Traditional Chinese Medicine, Yijishan Hospital of Wannan Medical College, Wuhu, China; e Department of Gastroenterology, China-Japan Friendship Hospital, Chaoyang District, Beijing, China; f Department of Clinical Medicine, College of Veterinary Medicine, China Agricultural University, Beijing, China

**Keywords:** Precancerous gastric lesions, Machine learning, Screening tool, Symptom and lifestyle

## Abstract

**Background:**

Precancerous gastric lesions (PLGC) are a critical stage in gastric cancer progression, where timely intervention can substantially reduce mortality. However, current screening strategies are predominantly endoscopic, which are invasive, costly, and often inaccessible in resource-limited settings. We aimed to develop and validate an interpretable machine learning model for non-invasive PLGC screening using symptom and lifestyle data.

**Methods:**

In this multicentre study, we enrolled eligible adult participants undergoing or scheduled to undergo upper gastrointestinal endoscopy with no prior diagnosis of malignancy. The development cohort comprised 1034 participants recruited at two hospitals between Nov 16, 2022, and Apr 7, 2023. Symptom and lifestyle data from this cohort were used to construct the development dataset, which was randomly split into a training set (n = 620), an internal validation set (n = 207), and a hold-out test set (n = 207). External performance was assessed in a retrospective hospital-based cohort from four additional hospitals (n = 630; May 21, 2018 to Jul 30, 2023) and a prospective community-based cohort from 32 screening sites (n = 847; June 21, 2023, to Nov 7, 2023). We developed a stacking ensemble model to predict the primary outcome (presence of PLGC) by integrating seven base learners (Gaussian Naïve Bayes, Logistic Regression, K-Nearest Neighbours, Gradient Boosting Classifier, eXtreme Gradient Boosting, Random Forest, Adaptive Boosting) and applied Shapley Additive Explanations (SHAP) for clinical interpretability. Model performance was compared with guideline-based screening strategies from the *Chinese Guidelines for Gastric Cancer Screening and Early Diagnosis and Treatment* and the *British Society of Gastroenterology gastric cancer risk guidance*, using the area under the receiver operating characteristic curve (AUC; 95% CI), sensitivity, specificity, positive predictive value, and negative predictive value.

**Findings:**

In total, 2511 participants (male: n = 871, 34.7%; female: n = 1640, 65.3%) were included. The primary outcome, PLGC, was present in 509 of 1034 participants (49.2%) in the development cohort, in 331 of 630 participants (52.5%) in the retrospective validation cohort, and in 312 of 847 participants (36.8%) in the prospective validation cohort. The model showed robust performance for non-invasive PLGC screening, with AUCs of 0.82 (95% CI: 0.77–0.87) in the internal hold-out test set, 0.80 (95% CI: 0.78–0.82) in the external retrospective validation set, and 0.79 (95% CI: 0.77–0.81) in the prospective validation set. With AUC improvements of 0.18–0.35, our model exceeded both guideline-based strategies across all datasets (internal hold-out test: 0.82 (95% CI: 0.77–0.87) vs. 0.47 (95% CI: 0.42–0.53)/0.48 (95% CI: 0.42–0.53); external retrospective validation: 0.80 (95% CI: 0.78–0.82) vs. 0.62 (95% CI: 0.60–0.64)/0.58 (95% CI: 0.55–0.60); prospective validation: 0.79 (95% CI: 0.77–0.81) vs. 0.57 (95% CI: 0.54–0.59)/0.52 (95% CI: 0.50–0.55); all p < 0.001). In a cost-effectiveness analysis, this translated into a 37.1% reduction in the average cost per detected PLGC case versus guideline-based tools. SHAP analysis further identified 15 key predictors, including *Helicobacter pylori* infection, age, and melaena.

**Interpretation:**

An interpretable machine learning model integrating symptom and lifestyle information, some of which were implicated by traditional medicine, achieved superior performance to guideline-based screening strategies for PLGC non-invasive screening in both hospital-based and community-based populations. However, the generalisability may be limited by the cohorts’ age and regional distribution; further studies should incorporate more non-invasive metrics to optimise the screening model and pursue broader external validation and real-world implementation.

**Funding:**

10.13039/501100001809National Natural Science Foundation of China. Innovation Team and Talents Cultivation Program of the National Administration of Traditional Chinese Medicine. Fundamental and Interdisciplinary Disciplines Breakthrough Plan of the Ministry of Education of China.

**Research in context**

**Evidence before this study**

Gastric cancer (GC) remains one of the most burdensome malignancies worldwide. We searched PubMed up to August 10, 2025, using the following terms: (“precancerous gastric lesion” OR “gastric precancerous lesion” OR “premalignant gastric lesion” OR “gastric premalignant lesion”) AND (“screening” OR “diagnosis” OR “classification”) AND (“machine learning” OR “artificial intelligence”) AND (“noninvasive” OR “non-invasive”). Only 11 relevant studies were identified. Previous non-invasive screening strategies for precancerous gastric lesions (PLGC) can be broadly grouped into three major categories. Some studies have developed rule-based or guideline-derived risk scoring models that depend on a limited number of questionnaire or clinical variables, which restrict their predictive performance. Others have explored imaging-based artificial intelligence (AI) methods—such as tongue-image or hyperspectral analysis—that require specialised equipment and trained operators, while the biological relevance between image-derived features and gastric pathology remains uncertain. In addition, several studies have focused on biochemical or metabolomic assays, including salivary or urinary biomarker testing, which rely on laboratory infrastructure and are relatively costly. Collectively, current research still lacks an interpretable and externally validated non-invasive screening model that relies solely on symptom and lifestyle information.

**Added value of this study**

To our knowledge, this study is the first to develop and externally validate an interpretable machine learning model based solely on symptom and lifestyle data for non-invasive screening of precancerous gastric lesions. This model was evaluated and validated in multicentre cohorts encompassing both hospital-based and community-based populations, demonstrating superior performance compared to guideline-based screening strategies. This provides a solution for implementing pre-endoscopic risk stratification in large-scale populations. Furthermore, through Shapley Additive Explanations-based interpretability analysis, in addition to classical risk factors such as *Helicobacter pylori* infection and age, several clinical and lifestyle features with potential for PLGC screening were identified, including drinking habits, dietary preferences, and gastrointestinal symptoms (e.g., belching), providing physicians with a transparent and interpretable decision-making reference.

**Implications of all the available evidence**

Given the high prevalence of precancerous gastric lesions and the substantial economic burden of invasive endoscopy screening strategies, our study highlights the value of non-invasive PLGC screening based on a combination of conventional risk factors and lifestyle information, potentially serving as a valuable complement to existing endoscopic screening strategies. By improving the precision and targeting of endoscopic examinations, this approach could potentially enhance screening efficiency and reduce healthcare costs, particularly in resource-limited settings. However, the generalisability may be limited by the cohorts’ age and regional distribution. Future research integrating additional non-invasive modalities (e.g., imaging data) may further improve model accuracy, and future validation studies across cohorts in more regions would further enhance the model generalisability.

## Introduction

Gastric cancer (GC) is the fifth most commonly diagnosed malignancy and the third leading cause of cancer-related mortality worldwide,^1^^,^^2^ with disproportionately high incidence in Eastern Asia, Eastern Europe, and Central Europe.^3^ Despite advances in surgical techniques,^4^ chemotherapy regimens,^5^ and targeted therapies,^6^ prognosis for advanced GC remains poor, with persistently low five-year survival rates. The substantial clinical and socioeconomic burdens underscore the urgent need for strategies that enable earlier detection. Thus, early and precise identification of high-risk populations prior to GC onset is of paramount importance in reducing both the GC incidence and mortality.

Precancerous gastric lesions (PLGC)—including chronic atrophic gastritis, intestinal metaplasia, and dysplasia—represent pathological alterations in the gastric mucosa that precede intestinal-type gastric malignancy.^7^, ^8^, ^9^ Identification and management of PLGC offer a critical opportunity to interrupt the carcinogenic cascade and prevent progression to GC. In clinical practice, endoscopic examination serves as the most widely utilised strategy for screening and diagnosing PLGC, offering high diagnostic accuracy but posing substantial barriers to widespread implementation. Beyond procedural costs, expenses related to equipment maintenance and specialist training increase the financial burden, and the invasive nature of endoscopy often deters patient compliance due to discomfort and procedure-related anxiety. In resource-limited settings, such as rural and economically underdeveloped regions, shortages of skilled gastroenterologists and specialised equipment exacerbate access disparities.^10^, ^11^, ^12^ Consequently, many high-risk individuals face delayed diagnosis or forgo screening entirely, increasing the likelihood of advanced-stage presentation.

Non-invasive screening methods offer a promising solution to address the issue.^13^, ^14^, ^15^ Readily obtainable data—such as symptom profiles (e.g., lassitude, nausea, acid regurgitation, dry mouth) and lifestyle factors (e.g., smoking, alcohol consumption, high-salt diets)—can be leveraged for early risk assessment without invasive procedures. Such approaches not only reduce patient discomfort but also expand coverage, enabling the identification of at-risk individuals, thereby improving early detection rates and enhancing quality of life. In parallel, machine learning (ML) technologies are increasingly applied across healthcare for multivariable analysis and complex pattern recognition.^16^, ^17^, ^18^, ^19^, ^20^ ML can uncover subtle, non-linear associations between clinical, lifestyle, and genetic factors that traditional statistical methods may overlook. For instance, ML has been successfully applied to predict hepatic inflammation in chronic hepatitis B^21^ and diagnose heart disease in patients with diabetes.^22^ By integrating routinely collected patient data, ML models can deliver personalised, automated risk assessments and update predictions as new information becomes available,^23^, ^24^, ^25^, ^26^, ^27^, ^28^, ^29^ enabling timely, targeted interventions and more efficient allocation of healthcare resources. Accordingly, several ML-driven approaches were established for gastric precancerous and cancer lesion detection.^30^, ^31^, ^32^ However, there is still a lack of an interpretable ML model suitable for non-invasive screening of PLGC, integrating symptom profiles and lifestyle factors.

In this study, we developed an advanced, interpretable machine learning–based model for the non-invasive screening of PLGC, leveraging symptom and lifestyle data collected from multi-centre cohorts across diverse regions in China. Unlike previous approaches, our model combines rigorous feature selection with Shapley Additive Explanations (SHAP) to enhance interpretability, ensuring that risk predictions are both efficient and clinically transparent. The performance of our model was evaluated by benchmarking multiple algorithms in independent external cohorts. In summary, our study offers a new perspective for the PLGC non-invasive screening and might refine current gastric cancer risk early-warning strategies.

## Methods

### Study population

We conducted a multicentre cohort study comprising a development cohort and two independent external validation cohorts in China. The development cohort included consecutive participants recruited from two hospitals in Fujian Province between Nov 16, 2022, and Apr 7, 2023. External validation used two prespecified datasets that were completely independent of model training: (1) a retrospective hospital-based cohort from four tertiary hospitals in Beijing and Anhui Province between May 21, 2018 and Jul 30, 2023; and (2) a prospective community-based cohort recruited at 32 community screening sites in Fujian Province between Jun 21 and Nov 7, 2023 (Supplementary Table S2 and S3). Together, the participating sites spanned northern China (Beijing), central China (Anhui Province), and southern China (Fujian Province), capturing heterogeneity in socioeconomic context, dietary patterns, environmental exposures, and access to health care. This multisite design was intended to improve the representativeness of the study population and to strengthen the external validity and potential generalisability of the model.

### Sample size determination

At the design stage, the prevalence of PLGC in the target screening population was uncertain. We therefore referred to prior epidemiological reports from China and other Asian regions indicating a PLGC prevalence of approximately 20–30%, and adopted a working prevalence of p = 0.25 for sample size planning.^33^ To ensure adequate model stability and limit overfitting, we followed the events-per-variable (EPV) principle tailored to penalised logistic modelling and feature selection, targeting EPV = 15.^34^ With k = 31 candidate predictors, the required total sample size was

$$N=\frac{k\;\times \;EPV}{p}=\frac{31\;\times \;15}{0.25}=1860.$$

Our study ultimately enrolled N = 2511 participants, far exceeding this target. This adheres to contemporary recommendations that complement the traditional EPV rule with validation- and shrinkage-oriented considerations in prediction modelling,^35^ supporting the adequacy and scientific rationale of our sample size for model development and validation.

### Ethics statement

The study protocol was reviewed and approved by the Ethics Committee of Fujian Medical University (Approval No.: 86&12, 2022; Approval No.: 120, 2022), the Scientific Research and New Technology Institutional Review Board of Yijishan Hospital of Wannan Medical College (Approval No. 3, 2020), the Institutional Review Board of Tsinghua University (Approval No.: 0069, 2020), the Clinical Research Ethics Committee of China–Japan Friendship Hospital (Approval No.: 2020-10-K07, 2020; Approval No.: 2023-KY-174, 2023), and all participants provided written informed consent prior to enrolment. All procedures followed were in accordance with the ethical standards of the responsible committee on human experimentation (institutional and national) and with the Helsinki Declaration of 1975, as revised in 2008.

### Patient enrolment

Eligible participants met the following inclusion criteria: (i) age 18–70 years; (ii) availability of gastroscopy results within the preceding three months or scheduled gastroscopy within the following three months; (iii) no prior diagnosis of malignancy; (iv) local residency with willingness to participate in follow-up; and (v) provision of informed consent. Exclusion criteria comprised: (i) current or previous cancer diagnosis; (ii) contraindications to endoscopic examination; (iii) pregnancy, planned pregnancy, or lactation; (iv) presence of major cardiovascular, pulmonary, renal, endocrine, neurological, or haematological disorders; (v) clinically diagnosed mental illness; and (vi) unwillingness or inability to comply with study requirements.

### Data collection

Comprehensive data were collected through structured interviews conducted by trained healthcare professionals or electronically administered questionnaires, encompassing demographic variables, symptom profiles, and lifestyle behaviours.

Demographics. Core demographic information included age, sex, and family history of gastric cancer. Age was recorded to evaluate its association with lesion prevalence and disease progression across strata, while sex was analysed to explore potential disparities in presentation and outcomes. Sex was self-reported in the structured questionnaire according to the binary categories (male/female) used in the hospital registration system and was treated as a biological variable; gender identity was not separately assessed. Family history was systematically documented to capture hereditary predisposition, particularly in individuals with affected first-degree relatives.

Clinical symptoms. Symptom data were elicited through detailed interviews, and subsequently categorised into four domains: (i) gastric symptoms (e.g., abdominal pain, bloating, nausea, belching); (ii) oral symptoms (e.g., halitosis, bitter taste, dry mouth, oral ulcers); (iii) bowel symptoms (e.g., constipation, diarrhoea, melaena); and (iv) systemic changes, including appetite loss, fatigue, and weakness. This structured classification provided a comprehensive framework to examine symptom–disease associations and their contribution to precancerous lesion risk. In designing the symptom items, we incorporated both conventional gastroenterological manifestations and symptom constructs that are routinely used in traditional Chinese medicine (TCM, e.g., halitosis, bitter taste in the mouth, dry mouth, aversion to cold with cold limbs, and preferences for warm or cold food), all of which were operationalised as standardised questionnaire variables.

Lifestyle factors. Lifestyle variables were assessed with a focus on dietary habits, smoking, and alcohol consumption. Particular attention was given to the frequency of consumption of oily, salty, and spicy foods, given their known role in gastric carcinogenesis. Smoking and alcohol use were documented in detail to further elucidate their contribution to lesion development and progression.

To ensure methodological transparency and reproducibility, the dataset was preprocessed to exclude participants with missing values or implausible data (e.g., age recorded as an identity number). This process reduced the primary set from 1135 to 1034 individuals, the external validation set 1 from 751 to 630 individuals, and the external validation set 2 from 1000 to 847 individuals, forming the final analytical sample. The development dataset was divided into training (60%), internal validation (20%), and internal hold-out test (20%) subsets using stratified random sampling, maintaining consistent class proportions across all partitions. Feature selection—including L1-regularised logistic regression and SHAP-based importance ranking—was conducted strictly within the training set to avoid information leakage. The internal validation set was used solely for hyperparameter tuning, while the internal test set was reserved as an untouched hold-out set for internal performance assessment. Finally, an independent external validation dataset was used to evaluate model generalisability.

### Endoscopic examination

All participants underwent esophagogastroduodenoscopy, performed by two independent experienced gastroenterologists. Biopsy specimens were histologically classified as normal, superficial gastritis, chronic atrophic gastritis, intestinal metaplasia, or intraepithelial neoplasia, with the most severe lesion used as the diagnostic endpoint. *Helicobacter pylori* infection was assessed by enzyme-linked immunosorbent assay (ELISA) for plasma IgG. To minimise bias, all endoscopic procedures were performed within a three-month window of questionnaire collection, and endoscopists were blinded to participants’ symptom profiles and medical histories.

### Machine learning-based risk models

The overall modelling framework is illustrated in Supplementary Materials (Supplementary Figure S1), which comprises four consecutive stages: data collection and preprocessing, feature selection, model construction, and performance evaluation with interpretability analysis. In the first stage, structured questionnaires were designed to capture four information domains—demographics, overall symptoms, oral symptoms and gastric symptoms. Raw entries were parsed into text and numerical variables, which were encoded and standardised. A complete-case analysis approach was adopted, whereby systematic outlier detection was conducted, and any samples containing missing data or outliers were removed. The processed dataset was stratified into training, internal validation, and internal hold-out for model development.

During feature selection, Logistic Regression (LR)-based SHAP importance ranking, Area Under the Curve (AUC)–feature curve–guided elimination, and correlation analysis were jointly applied to remove redundant or highly collinear variables while retaining clinically meaningful predictors.

The modelling stage adopted a stacking ensemble architecture: multiple base learners generated out-of-fold predictions that were used by a meta-learner, with hyperparameter optimisation conducted on the validation set to stabilise performance before model locking. Finally, model performance was evaluated using area under the receiver operating characteristic curve (AUC), sensitivity (Se), specificity (Sp), positive predictive value (PPV), and negative predictive value (NPV), while interpretability was examined at both global and local levels via SHAP-based importance plots and case-specific explanations, providing transparent and clinically auditable insights into model reasoning.^35^, ^36^, ^37^

We benchmarked seven widely used machine learning algorithms with complementary strengths. Gaussian Naïve Bayes (GNB) is a probabilistic classifier that assumes conditional independence among features, offering computational efficiency and robustness in high-dimensional settings.^38^ LR, a classical and interpretable baseline, models the log-odds of PLGC risk and provides well-calibrated probability estimates.^39^ K-Nearest Neighbours (KNN) classifies cases based on feature similarity, enabling flexible decision boundaries in heterogeneous symptom profiles. Gradient Boosting Classifier (GBC) and eXtreme Gradient Boosting (XGB) are boosting-based methods that iteratively refine weak learners to capture complex, non-linear associations^40^; XGB is optimised for efficiency on large-scale, high-dimensional datasets.^41^ Random Forest (RF) aggregates multiple decision trees, reducing variance and overfitting while preserving interpretability through feature importance measures.^42^ Adaptive Boosting (ADB) sequentially reweights misclassified cases, enhancing sensitivity for hard-to-classify patients.^43^

To mitigate overfitting risk and enhance model generalisability, we implemented regularisation strategies combined with systematic grid search for hyperparameter optimisation across all models. Specifically, LR was optimised for regularisation strength (C: search range 1e-3 to 1e3, final value 1.0), penalty type (L1/L2, final L2), and maximum iterations (100–1000, final 500); GBC was tuned for maximum tree depth (1–9, final 1), learning rate (1e-3 to 1e3, final 0.2), and number of estimators (10–1000, final 300); RF was optimised for maximum tree depth (1–9 and None, final None) and number of estimators (10–1000, final 50); XGB was adjusted for maximum tree depth (1–9, final 3) and learning rate (1e-3 to 1e3, final 0.2); ADB was optimised for number of estimators (10–1000, final 800) and learning rate (1e-3 to 1e3, final 2.0); KNN was tuned for number of neighbours (1–9, final 3); while GNB used default parameters. All hyperparameters were optimised by training on the training set and evaluating on the internal validation set, with the configuration achieving the highest AUC selected as optimal. These comprehensive measures ensured robust and generalisable risk predictions.

While each of these algorithms provides unique advantages, none alone is sufficient to fully capture the complex and heterogeneous patterns underlying PLGC risk. To harness their complementary strengths, we further implemented two ensemble strategies. Soft voting aggregated the predicted probabilities from individual classifiers, with the final prediction determined by weighted averaging across models. Stacking employed a meta-learner trained on the probabilistic outputs of the base models to integrate their predictions. Both ensemble approaches were designed to leverage complementary model strengths and to improve diagnostic stability across datasets.

For the Stacking hyperparameter, we implemented a two-stage optimisation: first, we systematically evaluated all seven individual models as candidate meta-learners using default parameters and selected LR as the optimal meta-learner based on its superior performance; second, we performed grid search to optimise the meta-learner hyperparameters, including regularisation strength (1e-3 to 1e3, final 0.1), penalty type (L1/L2, final L1), and maximum iterations (100–1000, final 1000).

Model development and validation were reported in accordance with the TRIPOD+AI statement (Transparent Reporting of a multivariable prediction model for Individual Prognosis or Diagnosis with Artificial Intelligence).^44^

### Statistical analysis

A total of 31 candidate variables were initially included in model development, encompassing demographic characteristics, symptom domains, lifestyle factors, and *Helicobacter pylori* infection status. These variables were entered into nine machine learning algorithms to generate preliminary prediction models for PLGC.

To identify the most predictive features for model construction, we implemented a two-step feature selection pipeline. First, a LR model with L1 regularisation was pre-trained on the training set. Subsequently, SHAP analysis was applied to this pre-trained model using the training set to quantify and rank feature contributions. Leveraging this feature ranking, we systematically evaluated the impact of feature set size on model performance by testing subsets encompassing from the top 5 to all 31 features. This investigation revealed that models utilising between 15 and 20 features achieved an optimal performance. For the most parsimonious yet effective configuration, the top 15 features with the highest SHAP importance scores were retained for final model construction.

Model performance was evaluated using standard diagnostic metrics, including AUC, Se, Sp, PPV, NPV, positive likelihood ratio (LR_POS), and negative likelihood ratio (LR_NEG). Differences in AUC between other models and the stacking model were assessed using the DeLong test, with results reported as Z-statistic and corresponding p-values. Clinical utility was further examined by DCA, and calibration was assessed to evaluate agreement between predicted and observed probabilities.

We compared a guideline-based screening strategy with the proposed model-based strategy using a simplified per-encounter cost-effectiveness framework. Primary outcomes were: (1) the number of endoscopies per 10 000 individuals screened; (2) the number of PLGC cases detected per 10 000; and (3) the cost per case detected (CPCD), defined as total endoscopy expenditure divided by the number of true-positive PLGC cases. The analysis was parameterised using the observed PLGC prevalence and the sensitivity and specificity of each strategy in external validation set 2. We assumed a fixed unit cost per endoscopy; additional laboratory, imaging, adverse-event, and downstream treatment costs were not included. Full analytic expressions, symbol definitions, and modelling assumptions are provided in Supplementary Methods S1.

### Role of the funding source

The funder of the study had no role in study design, data collection, data analysis, data interpretation, or writing of the report. All authors had full access to all the data in the study and take responsibility for the integrity of the data and the accuracy of the data analysis. The decision to submit the manuscript for publication was made jointly by all authors, with SL taking overall responsibility for the submission.

## Results

### Patient characteristics

**Fig. 1:**
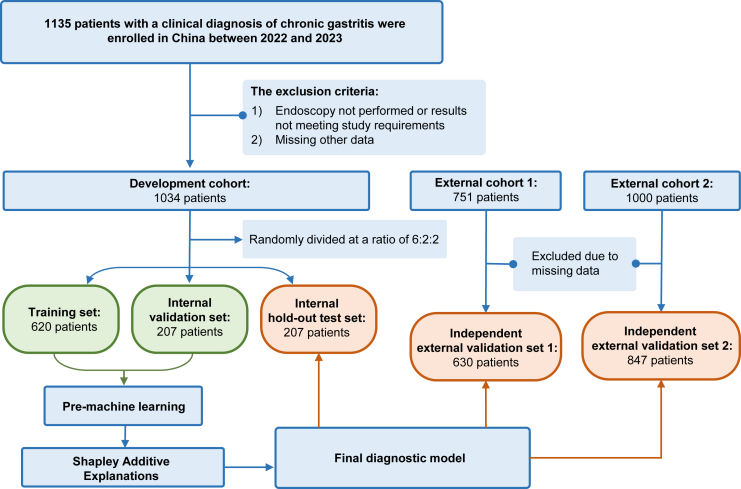
**Flow diagram of the study population and dataset partitioning.** This diagram shows how the primary development dataset and two independent external validation cohorts were constructed. Among 1135 enrolled patients with chronic gastritis, 1034 formed the primary dataset for model development after excluding individuals without qualifying endoscopy results or with missing key data. The primary dataset was split by stratified random sampling into a training set (n = 620), an internal validation set (n = 207), and an internal hold-out test set (n = 207). In addition, 1000 and 751 further patients constituted two external datasets; after excluding cases with missing data, these yielded two independent external validation cohorts (n = 630 and 847).

In total, 2511 participants (male: n = 871, 34.7%; female: n = 1640, 65.3%) were included. The primary outcome, PLGC, was present in 509 of 1034 participants (49.2%) in the development cohort, in 331 of 630 participants (52.5%) in the retrospective validation cohort, and in 312 of 847 participants (36.8%) in the prospective validation cohort. The development cohort consisted of symptom and lifestyle data collected at two hospitals between Nov 16, 2022 and Apr 7, 2023. It was randomly partitioned (6:2:2) into a training set (n = 620), an internal validation set (n = 207), and a hold-out test set (n = 207). External validation comprised: (i) a retrospective cohort from four additional hospitals (n = 630), and (ii) a prospective, community-based cohort from 32 screening sites (n = 847; Jun 21–Nov 7, 2023). The participant flow and dataset construction are summarised in Fig. 1.

Baseline characteristics of the five sets are summarised in Table 1. In the development dataset (training, internal validation, and internal hold-out test sets; n = 1034), the median age was approximately 60 years, and the proportion of male participants was 34.8% in the training and internal validation sets, 28.0% in the internal hold-out test set, and 19.4% and 47.6% in the external validation sets. Comprehensive symptom and lifestyle data were collected from all participants, covering variables such as pain type, gastrointestinal discomfort and dietary habits. In addition, *Helicobacter pylori* infection status was recorded (infection rates: 40.8%, 43.0%, 30.9%, 15.6%, and 33.6% in the five sets, respectively). This ensured a robust and diverse dataset for model development and validation.

**Table 1:** Baseline characteristics of the study sets.

	Training set (n = 620)	Internal validation set (n = 207)	Internal hold-out test set (n = 207)	Independent external validation set 1 (n = 630)	Independent external validation set 2 (n = 847)
Age (years, mean ± SD)	60.3 ± 9.1	61.0 ± 7.9	60.4 ± 9.1	55.4 ± 13.4	47.5 ± 11.7
Sex, n (%)					
Male	216 (34.8)	72 (34.8)	58 (28.0)	122 (19.4)	403 (47.6)
Female	404 (65.2)	135 (65.2)	149 (72.0)	508 (80.6)	444 (52.4)
Family history of gastric cancer, n (%)	29 (4.7)	4 (1.9)	8 (3.9)	112 (17.8)	99 (11.7)
Alcohol consumption, n (%)	2 (0.3)	0 (0.0)	0 (0.0)	1 (0.2)	13 (1.5)
Smoking, n (%)	73 (11.8)	25 (12.1)	25 (12.1)	21 (3.3)	123 (14.5)
Vague pain, n (%)	183 (29.5)	62 (30.0)	56 (27.1)	194 (30.8)	187 (22.1)
Distending pain, n (%)	44 (7.1)	20 (9.7)	12 (5.8)	176 (27.9)	118 (13.9)
Pricking pain, n (%)	59 (9.5)	30 (14.5)	25 (12.1)	41 (6.5)	37 (4.4)
Burning pain, n (%)	61 (9.8)	21 (10.1)	20 (9.7)	79 (12.5)	21 (2.5)
Abdominal distention, n (%)	147 (23.7)	64 (30.9)	60 (29.0)	352 (55.9)	229 (27.0)
Belching, n (%)	152 (24.5)	49 (23.7)	47 (22.7)	340 (54.0)	246 (29.0)
Acid regurgitation, n (%)	163 (26.3)	54 (26.1)	52 (25.1)	314 (49.8)	240 (28.3)
Dry mouth, n (%)	263 (42.4)	82 (39.6)	96 (46.4)	171 (27.1)	251 (29.6)
Bitter taste in the mouth, n (%)	259 (41.8)	77 (37.2)	85 (41.1)	202 (32.1)	165 (19.5)
Halitosis, n (%)	127 (20.5)	40 (19.3)	44 (21.3)	25 (4.0)	294 (34.7)
Dry stool, n (%)	91 (14.7)	28 (13.5)	39 (18.8)	92 (14.6)	103 (12.2)
Loose stool, n (%)	85 (13.7)	28 (13.5)	29 (14.0)	131 (20.8)	187 (22.1)
Sticky stool, n (%)	24 (3.9)	7 (3.4)	9 (4.3)	5 (0.8)	174 (20.5)
Black stool, n (%)	23 (3.7)	9 (4.3)	10 (4.8)	9 (1.4)	48 (5.7)
Aversion to cold with cold limbs, n (%)	104 (16.8)	43 (20.8)	46 (22.2)	127 (20.2)	199 (23.5)
Poor appetite, n (%)	30 (4.8)	8 (3.9)	8 (3.9)	89 (14.1)	54 (6.4)
Hypochondriac pain, n (%)	113 (18.2)	41 (19.8)	43 (20.8)	76 (12.1)	55 (6.5)
Water brash (Clear watery vomitus), n (%)	92 (14.8)	30 (14.5)	30 (14.5)	0 (0.0)	9 (1.1)
Lassitude, n (%)	104 (16.8)	44 (21.3)	34 (16.4)	138 (21.9)	166 (19.6)
Preference for warm food, n (%)	548 (88.4)	174 (84.1)	186 (89.9)	45 (7.1)	180 (21.3)
Preference for cold conditions, n (%)	118 (19.0)	36 (17.4)	37 (17.9)	137 (21.7)	104 (12.3)
Anaemia, n (%)	60 (9.7)	17 (8.2)	18 (8.7)	14 (2.2)	94 (11.1)
Nausea, n (%)	32 (5.2)	12 (5.8)	9 (4.3)	96 (15.2)	88 (10.4)
Pickled and smoked foods, n (%)	34 (5.5)	9 (4.3)	6 (2.9)	33 (5.2)	39 (4.6)
Fried foods, n (%)	19 (3.1)	10 (4.8)	4 (1.9)	35 (5.6)	127 (15.0)
*Helicobacter pylori* infection, n (%)	253 (40.8)	89 (43.0)	64 (30.9)	98 (15.6)	285 (33.6)

In total, 31 clinical variables were assessed for model construction, covering demographic factors, gastrointestinal and systemic symptoms, lifestyle behaviours, and *Helicobacter pylori* infection status: age, sex, family medical history, vague pain, distending pain, pricking pain, burning pain, abdominal distention, belching, acid regurgitation, dry mouth, bitter taste in the mouth, halitosis, dry stool, loose stool, sticky stool, black stool, cold limbs, poor appetite, nausea, hypochondriac pain, water brash, lassitude, preference for warm food, preference for cold food, pickled and smoked foods, fried foods, alcohol consumption, smoking, *Helicobacter pylori* infection, and anaemia.

### Diagnostic performance

Among all candidate algorithms, model comparison and selection were first conducted within the internal validation set of the development data. The stacking ensemble was identified as the optimal model based on its superior AUC and calibration performance in this internal validation phase, while the independent test and external validation datasets were used solely for final performance evaluation.

**Fig. 2:**
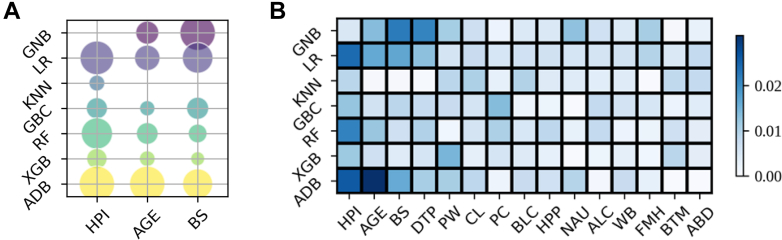
**Feature importance analysis across seven machine learning models.** (A) Bubble plot of Shapley Additive Explanations (SHAP) values for the three most influential features in each model. Circle size represents the mean absolute SHAP value (larger circles indicate greater importance), and colour distinguishes different models. (B) Heatmap of the 15 most important features across all seven models. Each cell shows the normalised mean absolute SHAP value for a given feature–model pair; darker blue indicates higher feature importance (scale bar on the right). ABD = Abdominal Distention. ADB = Adaptive Boosting. ALC = Alcohol Consumption. BLC = Belching. BS = Black Stool. BTM = Bitter Taste in the Mouth. CL = Cold Limbs. DTP = Distending Pain. FMH = Family Medical History. GBC = Gradient Boosting Classifier. GNB = Gaussian Naive Bayes. HPI = *Helicobacterpylori* Infection. HPP = Hypochondriac Pain. KNN = K-nearest neighbours. LR = Logistic Regression. NAU = Nausea. PC = Preference for Cold Food. PW = Preference for Warm Food. RF = Random Forest. WB = Water Brash (Clear Watery Vomitus). XGB = eXtreme Gradient Boosting.

After determining the optimal modelling framework, we further examined the relationship between the number of selected features and model performance. As features were sequentially added in descending order of SHAP importance, the AUC increased up to approximately 15 features and then plateaued, indicating that this number of variables offered the best trade-off between predictive accuracy and parsimony. These top-ranked features were subsequently incorporated into the final stacking model (Fig. 2). These features included *Helicobacter pylori* infection, age, black stool, distending pain, preference for warmth, cold limbs, preference for cold food, belching, hypochondriac pain, nausea, alcohol consumption, water brash, family medical history, bitter taste in the mouth, and abdominal distention. Expectedly, *Helicobacter pylori* infection, age and family medical history are well-documented as canonical risk factors for gastric cancer.

**Fig. 3:**
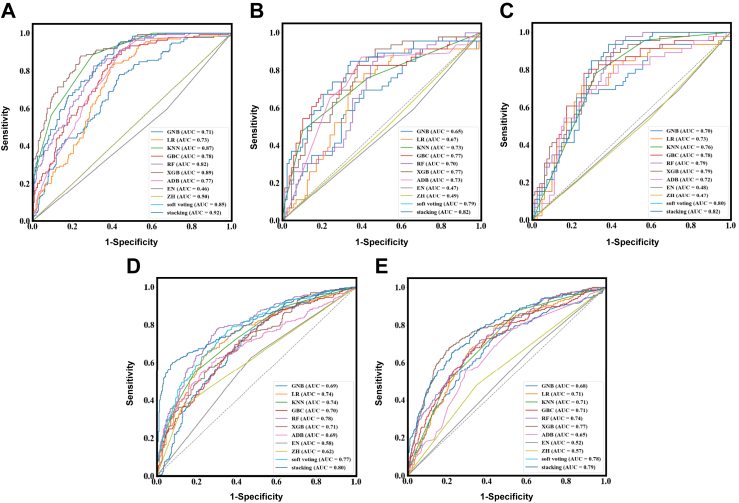
**Diagnostic performance of machine learning models for PLGC risk prediction across five sets.** (A) Training set. (B) Internal validation set. (C) Internal hold-out test set. (D) Independent external validation set 1. (E) Independent external validation set 2. Each coloured line represents one model or guideline. GBC = Gradient Boosting Classifier. RF = Random Forest. XGB = eXtreme Gradient Boosting. ADB = Adaptive Boosting. GNB = Gaussian Naive Bayes. LR = Logistic Regression. KNN = K-Nearest Neighbours. ZH = Chinese Guidelines for Gastric Cancer Screening and Early Diagnosis and Treatment. EN = British Society of Gastroenterology Guidelines for the Diagnosis of Gastric Cancer Risk. PLGC = Precancerous Gastric Lesions. AUC = Area Under the Receiver Operating Characteristic Curve.

Across the training, internal validation, internal hold-out test, and two external validation sets, AUC values of the nine models ranged from 0.71 to 0.92, 0.65 to 0.82, 0.70 to 0.82, 0.69 to 0.80, and 0.65 to 0.79, respectively (Fig. 3). The stacking ensemble consistently achieved the best overall performance, yielding the highest AUC in all sets. Statistical comparisons using DeLong's test against the stacking model revealed significant performance differences in multiple datasets (all p < 0.05), confirming its superiority over individual models (see Table 2 for detailed Z-statistics and p-values). Soft voting produced competitive results but remained inferior to stacking. In contrast, rule-based models derived from the Chinese Guidelines for Gastric Cancer Screening and Early Diagnosis and Treatment (ZH) and the British Society of Gastroenterology Guidelines for the Diagnosis of Gastric Cancer Risk (EN) performed poorly, with AUC values approximating 0.5 across all datasets; accordingly, the stacking model improved AUC by 0.18–0.35 (average 50.5% relative increase) over the guideline-based strategies (Table 2).

**Table 2:** Diagnostic performance of the machine learning models for diagnosing PLGC.

	Specificity (95% CI)	Sensitivity (95% CI)	PPV (95% CI)	NPV (95% CI)	LR_POS (95% CI)	LR_NEG (95% CI)	AUC (95% CI)	Z	P value
Training set									
GNB	0.58 (0.48–0.68)	0.70 (0.65–0.74)	0.30 (0.24–0.36)	0.88 (0.84–0.91)	1.93 (1.52–2.41)	0.60 (0.46–0.75)	0.71 (0.67–0.75)	10.20	<0.0001
LR	0.53 (0.47–0.59)	0.80 (0.75–0.85)	0.71 (0.65–0.78)	0.65 (0.60–0.70)	2.68 (2.10–3.59)	0.59 (0.51–0.67)	0.73 (0.70–0.77)	11.80	<0.0001
KNN	0.77 (0.69–0.83)	0.79 (0.75–0.83)	0.56 (0.50–0.64)	0.91 (0.87–0.94)	3.69 (3.01–4.66)	0.30 (0.21–0.38)	0.87 (0.85–0.89)	2.70	0.007
GBC	0.66 (0.57–0.74)	0.74 (0.70–0.78)	0.44 (0.37–0.51)	0.88 (0.84–0.91)	2.59 (2.10–3.16)	0.45 (0.34–0.57)	0.78 (0.75–0.81)	7.59	<0.0001
RF	0.86 (0.76–0.95)	0.70 (0.67–0.74)	0.24 (0.18–0.30)	0.98 (0.96–0.99)	2.92 (2.45–3.45)	0.19 (0.07–0.33)	0.82 (0.79–0.85)	5.70	<0.0001
XGB	0.79 (0.72–0.85)	0.82 (0.78–0.86)	0.64 (0.57–0.71)	0.91 (0.87–0.93)	4.43 (3.56–5.68)	0.26 (0.18–0.34)	0.89 (0.87–0.91)	1.99	0.047
ADB	0.57 (0.50–0.63)	0.79 (0.75–0.83)	0.64 (0.57–0.71)	0.73 (0.69–0.78)	2.69 (2.15–3.49)	0.55 (0.46–0.64)	0.77 (0.74–0.80)	6.16	<0.0001
EN	0.32 (0.27–0.37)	0.60 (0.52–0.66)	0.59 (0.52–0.66)	0.33 (0.28–0.38)	0.80 (0.63–1.01)	1.13 (0.99–1.32)	0.46 (0.43–0.49)	14.80	<0.0001
ZH	0.35 (0.30–0.41)	0.65 (0.59–0.70)	0.53 (0.46–0.61)	0.47 (0.41–0.52)	1.00 (0.79–1.26)	1.00 (0.89–1.13)	0.50 (0.46–0.53)	13.70	<0.0001
Soft voting	0.76 (0.68–0.84)	0.75 (0.71–0.79)	0.43 (0.36–0.49)	0.93 (0.90–0.95)	3.03 (2.52–3.68)	0.32 (0.21–0.43)	0.85 (0.82–0.87)	6.30	<0.0001
Stacking	0.67 (0.61–0.73)	0.94 (0.91–0.97)	0.91 (0.87–0.95)	0.76 (0.71–0.80)	10.97 (7.42–19.47)	0.35 (0.29–0.41)	0.92 (0.90–0.94)	–	–
Internal validation set									
GNB	0.56 (0.36–0.76)	0.69 (0.62–0.76)	0.22 (0.12–0.32)	0.91 (0.86–0.96)	1.79 (1.09–2.69)	0.64 (0.34–0.95)	0.65 (0.58–0.71)	3.99	<0.0001
LR	0.48 (0.37–0.59)	0.76 (0.67–0.84)	0.59 (0.46–0.71)	0.67 (0.58–0.75)	1.98 (1.32–3.09)	0.68 (0.53–0.86)	0.67 (0.61–0.74)	3.90	<0.0001
KNN	0.71 (0.57–0.84)	0.77 (0.70–0.83)	0.48 (0.35–0.60)	0.90 (0.84–0.95)	3.05 (2.13–4.37)	0.38 (0.21–0.57)	0.73 (0.67–0.79)	2.17	0.030
GBC	0.74 (0.60–0.86)	0.79 (0.72–0.85)	0.54 (0.42–0.66)	0.90 (0.84–0.95)	3.50 (2.51–5.20)	0.34 (0.18–0.50)	0.77 (0.71–0.82)	1.32	0.19
RF	0.69 (0.44–0.90)	0.69 (0.62–0.76)	0.20 (0.10–0.29)	0.95 (0.91–0.99)	2.26 (1.42–3.23)	0.44 (0.14–0.80)	0.70 (0.64–0.76)	3.62	<0.0001
XGB	0.67 (0.53–0.80)	0.76 (0.69–0.83)	0.48 (0.36–0.60)	0.88 (0.81–0.93)	2.81 (1.96–4.03)	0.44 (0.26–0.64)	0.77 (0.71–0.82)	1.43	0.15
ADB	0.59 (0.48–0.70)	0.81 (0.73–0.88)	0.65 (0.53–0.77)	0.76 (0.68–0.83)	3.05 (2.10–4.88)	0.51 (0.36–0.66)	0.74 (0.68–0.80)	1.47	0.17
EN	0.34 (0.26–0.43)	0.59 (0.47–0.70)	0.59 (0.47–0.70)	0.34 (0.25–0.43)	0.84 (0.58–1.22)	1.11 (0.89–1.44)	0.47 (0.41–0.52)	3.13	0.002
ZH	0.35 (0.25–0.45)	0.62 (0.52–0.72)	0.49 (0.37–0.61)	0.48 (0.39–0.57)	0.93 (0.61–1.39)	1.04 (0.84–1.31)	0.49 (0.43–0.55)	2.86	0.004
Soft voting	0.77 (0.61–0.91)	0.74 (0.67–0.81)	0.37 (0.25–0.49)	0.94 (0.90–0.98)	2.98 (2.20–4.19)	0.31 (0.12–0.53)	0.79 (0.73–0.84)	1.10	0.27
Stacking	0.80 (0.63–0.94)	0.74 (0.67–0.81)	0.35 (0.23–0.48)	0.95 (0.91–0.99)	3.04 (2.17–4.30)	0.27 (0.08–0.51)	0.82 (0.77–0.86)	–	–
Internal hold-out test set									
GNB	0.54 (0.38–0.69)	0.71 (0.61–0.80)	0.43 (0.30–0.58)	0.79 (0.70–0.87)	1.87 (1.21–2.97)	0.65 (0.42–0.89)	0.70 (0.64–0.76)	2.54	0.011
LR	0.57 (0.35–0.79)	0.68 (0.59–0.77)	0.26 (0.14–0.39)	0.89 (0.82–0.95)	1.78 (1.03–2.78)	0.63 (0.32–0.98)	0.73 (0.67–0.79)	2.16	0.031
KNN	0.58 (0.41–0.74)	0.71 (0.63–0.81)	0.41 (0.27–0.57)	0.83 (0.75–0.90)	2.00 (1.31–3.18)	0.60 (0.37–0.85)	0.76 (0.71–0.81)	1.88	0.061
GBC	0.63 (0.48–0.77)	0.75 (0.66–0.84)	0.52 (0.39–0.66)	0.83 (0.74–0.90)	2.56 (1.69–4.12)	0.49 (0.30–0.70)	0.78 (0.72–0.83)	1.42	0.16
RF	0.64 (0.44–0.83)	0.71 (0.61–0.79)	0.35 (0.22–0.49)	0.89 (0.82–0.95)	2.18 (1.33–3.32)	0.51 (0.25–0.82)	0.79 (0.74–0.84)	1.58	0.11
XGB	0.61 (0.47–0.76)	0.76 (0.67–0.85)	0.54 (0.40–0.69)	0.80 (0.72–0.88)	2.50 (1.68–4.12)	0.52 (0.32–0.72)	0.79 (0.74–0.84)	1.22	0.22
ADB	0.61 (0.42–0.79)	0.71 (0.62–0.80)	0.37 (0.24–0.51)	0.86 (0.79–0.94)	2.07 (1.36–3.15)	0.56 (0.30–0.83)	0.72 (0.66–0.78)	2.69	0.007
EN	0.36 (0.26–0.46)	0.58 (0.42–0.75)	0.69 (0.55–0.81)	0.27 (0.17–0.36)	0.87 (0.55–1.54)	1.09 (0.81–1.55)	0.48 (0.42–0.53)	5.17	<0.0001
ZH	0.36 (0.24–0.48)	0.59 (0.46–0.73)	0.54 (0.39–0.69)	0.41 (0.30–0.51)	0.87 (0.55–1.39)	1.09 (0.83–1.48)	0.47 (0.42–0.53)	5.39	<0.0001
Soft voting	0.58 (0.41–0.74)	0.71 (0.63–0.80)	0.41 (0.28–0.56)	0.83 (0.74–0.90)	2.00 (1.32–3.10)	0.60 (0.35–0.84)	0.80 (0.74–0.84)	1.15	0.25
Stacking	0.64 (0.47–0.80)	0.75 (0.66–0.84)	0.50 (0.35–0.65)	0.84 (0.76–0.92)	2.53 (1.61–4.19)	0.48 (0.27–0.72)	0.82 (0.77–0.87)	–	–
Independent external validation set 1									
GNB	0.67 (0.62–0.72)	0.62 (0.57–0.67)	0.64 (0.60–0.69)	0.64 (0.59–0.69)	1.74 (1.49–2.05)	0.54 (0.45–0.65)	0.69 (0.67–0.72)	4.94	<0.0001
LR	0.77 (0.71–0.82)	0.59 (0.55–0.64)	0.48 (0.43–0.53)	0.84 (0.80–0.88)	1.88 (1.65–2.15)	0.39 (0.29–0.50)	0.74 (0.71–0.76)	3.59	<0.0001
KNN	0.89 (0.81–0.96)	0.52 (0.48–0.56)	0.19 (0.15–0.23)	0.97 (0.95–0.99)	1.85 (1.63–2.05)	0.22 (0.09–0.38)	0.74 (0.71–0.76)	3.74	<0.0001
GBC	0.71 (0.64–0.78)	0.55 (0.50–0.60)	0.39 (0.34–0.44)	0.82 (0.78–0.87)	1.57 (1.37–1.81)	0.53 (0.41–0.66)	0.70 (0.67–0.72)	5.94	<0.0001
RF	0.00 (0.00–0.00)	0.47 (0.43–0.52)	0.00 (0.00–0.00)	1.00 (1.00–1.00)	0.00 (0.00–0.00)	2.11 (1.94–2.30)	0.78 (0.76–0.81)	0.96	<0.0001
XGB	0.81 (0.74–0.88)	0.55 (0.50–0.59)	0.31 (0.26–0.36)	0.92 (0.89–0.95)	1.79 (1.57–2.01)	0.35 (0.22–0.48)	0.71 (0.68–0.73)	3.94	0.17
ADB	0.77 (0.71–0.82)	0.58 (0.53–0.63)	0.44 (0.39–0.50)	0.85 (0.81–0.89)	1.82 (1.59–2.09)	0.40 (0.30–0.52)	0.69 (0.67–0.72)	5.41	<0.0001
EN	0.60 (0.55–0.65)	0.56 (0.50–0.62)	0.63 (0.58–0.68)	0.53 (0.47–0.59)	1.36 (1.17–1.58)	0.72 (0.62–0.84)	0.58 (0.55–0.60)	8.66	<0.0001
ZH	0.76 (0.69–0.83)	0.55 (0.51–0.60)	0.37 (0.31–0.41)	0.87 (0.84–0.91)	1.70 (1.49–1.94)	0.43 (0.31–0.56)	0.62 (0.60–0.64)	7.84	<0.0001
Soft voting	0.85 (0.78–0.91)	0.55 (0.51–0.59)	0.30 (0.26–0.35)	0.94 (0.91–0.97)	1.87 (1.67–2.12)	0.28 (0.16–0.41)	0.77 (0.75–0.80)	2.05	0.043
Stacking	0.94 (0.91–0.97)	0.64 (0.60–0.69)	0.52 (0.46–0.57)	0.97 (0.94–0.98)	2.65 (2.33–3.03)	0.09 (0.04–0.14)	0.80 (0.78–0.82)	–	–
Independent external validation set 2									
GNB	0.47 (0.38–0.56)	0.77 (0.74–0.80)	0.24 (0.18–0.29)	0.91 (0.88–0.93)	2.03 (1.57–2.53)	0.69 (0.57–0.81)	0.68 (0.65–0.70)	6.58	<0.0001
LR	0.43 (0.38–0.49)	0.83 (0.80–0.86)	0.59 (0.52–0.65)	0.73 (0.69–0.76)	2.57 (2.07–3.22)	0.68 (0.61–0.75)	0.71 (0.69–0.73)	5.61	0.0003
KNN	0.47 (0.41–0.54)	0.82 (0.79–0.84)	0.50 (0.43–0.56)	0.80 (0.77–0.83)	2.57 (2.08–3.12)	0.65 (0.56–0.73)	0.71 (0.69–0.74)	6.89	0.0002
GBC	0.54 (0.45–0.62)	0.79 (0.75–0.81)	0.31 (0.26–0.37)	0.90 (0.88–0.93)	2.50 (2.04–3.07)	0.59 (0.48–0.69)	0.71 (0.69–0.74)	5.22	<0.0001
RF	0.68 (0.56–0.80)	0.77 (0.74–0.79)	0.16 (0.12–0.21)	0.97 (0.96–0.98)	2.91 (2.37–3.55)	0.41 (0.27–0.58)	0.74 (0.71–0.76)	4.33	0.34
XGB	0.58 (0.52–0.64)	0.86 (0.83–0.88)	0.61 (0.55–0.67)	0.84 (0.81–0.87)	4.01 (3.31–5.03)	0.50 (0.42–0.57)	0.77 (0.75–0.80)	1.38	0.0001
ADB	0.39 (0.34–0.45)	0.81 (0.77–0.83)	0.51 (0.45–0.58)	0.72 (0.69–0.75)	2.03 (1.64–2.51)	0.75 (0.68–0.82)	0.65 (0.62–0.67)	7.17	<0.0001
EN	0.28 (0.24–0.31)	0.76 (0.71–0.81)	0.68 (0.62–0.74)	0.36 (0.33–0.40)	1.16 (0.92–1.51)	0.95 (0.88–1.03)	0.52 (0.50–0.55)	10.60	<0.0001
ZH	0.33 (0.28–0.38)	0.78 (0.74–0.81)	0.48 (0.41–0.54)	0.65 (0.62–0.69)	1.48 (1.20–1.82)	0.86 (0.79–0.94)	0.57 (0.54–0.59)	9.20	<0.0001
Soft voting	0.63 (0.55–0.71)	0.81 (0.78–0.84)	0.40 (0.34–0.46)	0.92 (0.89–0.94)	3.31 (2.75–4.02)	0.46 (0.36–0.56)	0.78 (0.75–0.80)	2.02	0.043
Stacking	0.75 (0.64–0.87)	0.77 (0.74–0.80)	0.18 (0.13–0.22)	0.98 (0.97–0.99)	3.24 (2.69–3.92)	0.33 (0.18–0.47)	0.79 (0.77–0.81)	–	–

P value is calculated using the DeLong's test. Abbreviations: CI, confidence interval; PPV, positive predictive value; NPV, negative predictive value; LR_POS, positive likelihood ratio; LR_NEG, negative likelihood ratio; AUC, area under the curve; ADB, adaptive boosting; GBC, gradient boosting classifier; GNB, gaussian naive bayes; KNN, K-nearest neighbours; LR, logistic regression; RF, random forest; XGB, eXtreme Gradient Boosting; EN, British Society of Gastroenterology Guidelines for the Diagnosis of Gastric Cancer Risk; ZH, Chinese Guidelines for Gastric Cancer Screening and Early Diagnosis and Treatment.

The optimal probability cutoff for the stacking model was determined as 0.22 by maximising the Youden index (Youden index = sensitivity + specificity − 1) on the internal validation set, which identifies the point on the receiver operating characteristic (ROC) curve that best balances sensitivity and specificity. This unified cutoff was applied to all models for consistent comparison. At this threshold, comprehensive diagnostic performance metrics—including sensitivity, specificity, PPV, NPV, LR_POS, and LR_NEG—for both the stacking model and all base models across all sets are detailed in Table 2. Table 2 also presents the AUC values and the results of statistical comparisons (DeLong's test Z-statistics and p-values) for each model against the stacking model, further corroborating its superior diagnostic performance.

### Decision curve and calibration analysis

**Fig. 4:**
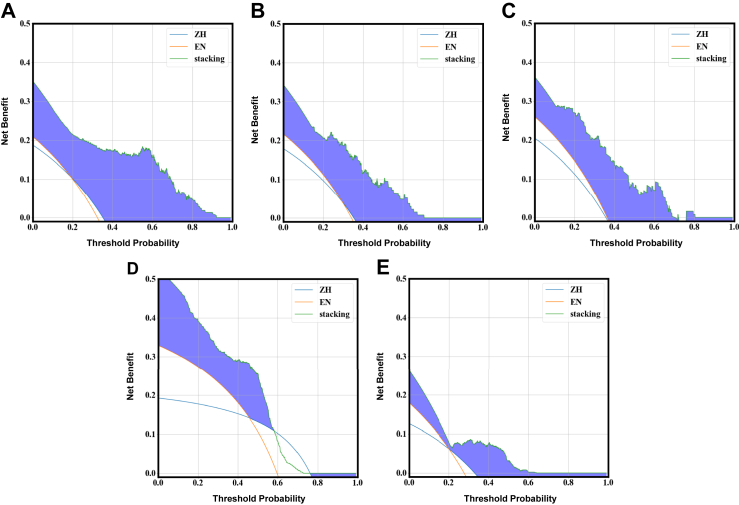
**Clinical utility of the stacking model for PLGC risk prediction evaluated using decision curve analysis (DCA) across five datasets.** (A) Training set. (B) Internal validation set. (C) Internal hold-out test set. (D) Independent external validation set 1. (E) Independent external validation set 2. Each curve represents the net benefit of using the stacking model or guideline-based strategies across a range of threshold probabilities. ZH = Chinese Guidelines for Gastric Cancer Screening and Early Diagnosis and Treatment. EN = British Society of Gastroenterology Guidelines for the Diagnosis of Gastric Cancer Risk. PLGC = Precancerous Gastric Lesions. DCA = Decision Curve Analysis.

Decision curve analysis demonstrated that the stacking model consistently provided the highest net clinical benefit across a broad range of threshold probabilities in the training (A), internal validation (B), internal hold-out test (C), external validation set 1 (D), and external validation set 2 (E) (Fig. 4). Across all thresholds, in all datasets, the stacking model yielded a greater net benefit than the rule-based strategies derived from the Chinese Guidelines for Gastric Cancer Screening and Early Diagnosis and Treatment (ZH) and the British Society of Gastroenterology Guidelines for the Diagnosis of Gastric Cancer Risk (EN). Specifically, the stacking model showed the largest clinical utility when the threshold probability was within the range of approximately 0.1–0.4, indicating that applying this model to identify high-risk individuals would achieve more true positives without increasing the number of unnecessary endoscopic examinations. These findings highlight its superior discriminative and clinical value for non-invasive screening.

Calibration curve analysis further showed good agreement between predicted and observed probabilities in the training set, with slightly reduced but acceptable calibration in the internal hold-out test and independent external validation sets. Together, these results indicate that the stacking model not only improves discrimination but also maintains reliable probability estimates, supporting its use as a clinically applicable screening tool.

### Cost-effectiveness analysis

To complement discriminative performance, we assessed cost efficiency from a healthcare-provider perspective using a simplified endoscopy-only framework. In the prospective external validation cohort (prevalence = 36.8%), assuming ¥800 per endoscopy, the model-based triage reduced the number of individuals requiring endoscopy by 37.9% relative to the guideline tool, primarily by suppressing false positives. This raised the positive predictive value by 23.8 percentage points while maintaining comparable sensitivity, and reduced the average cost per detected case by 37.1% (¥1246 vs. ¥1980). Together, these results indicate that—beyond improved discrimination—the proposed model materially lowers endoscopic workload and cost per detection while enhancing per-procedure yield, supporting its scalability as a non-invasive, low-cost pre-endoscopic screening tool. *(Illustrative economic comparison; downstream costs and health outcomes not included.)*

### Symptom patterns by temperature preference

We next examined heterogeneity by stratifying symptom profiles according to food-temperature preferences. To further explore the interpretability of the model, we analysed symptom patterns among participants with differing temperature preferences (Supplementary Figure S2). Participants diagnosed with PLGC who preferred cold food and disliked warmth were more likely to present with halitosis and bitter taste in the mouth. In contrast, individuals favouring warmth and avoiding cold exhibited a higher prevalence of dry mouth and vague pain, accompanied by a dietary inclination toward fried foods. Importantly, a habitual consumption of pickled and smoked foods was observed across both groups, suggesting a shared lifestyle factor that may exacerbate PLGC risk.

Although food-temperature preferences are not conventional biochemical or pathological indicators, their distribution patterns revealed distinct physiological tendencies among participants. Individuals with higher preference for warm food scores frequently reported habitual intake of hot food or beverages, which has been associated with repeated gastric mucosal stimulation and epithelial regeneration in previous studies.^45^^,^^46^ In contrast, participants with stronger preference for cold food tendencies often exhibited irregular meal timing and higher consumption of cold or processed foods, conditions linked to reduced gastric motility and altered mucosal perfusion.^47^^,^^48^

### Regional symptom patterns

To investigate geographical heterogeneity, we analysed symptom distributions among participants from different regions (Supplementary Figure S3). The regional distribution of participants is shown in Supplementary Figure S3A, while Supplementary Figure S3B depicts radar charts summarising representative PLGC-related features across four major regions. Although advanced age was a common risk factor in all sets, the characteristic patterns of PLGC varied substantially by region. In the Putian set, risk was predominantly associated with vague pain and abdominal distention. In Fuzhou, *Helicobacter pylori* infection emerged as a dominant feature. In Beijing, risk prediction was strongly linked to vague pain and family medical history, whereas in Wuhu, risk-related features were concentrated in *Helicobacter pylori* infection, preference for cold food and bitter taste in the mouth.

To provide a more granular view, Supplementary Figure S4 illustrates the distribution of the top 15 PLGC-associated features within each region. These findings reveal clear geographical patterns in risk characteristics, offering robust evidence to support the development of region-specific early screening strategies for PLGC.

## Discussion

This study develops and validates an interpretable ML model for screening PLGC using a large-scale dataset of patient symptoms and lifestyle information. By incorporating the SHAP method, the model achieves both high predictive performance and enhanced interpretability, enabling clinicians to quantify the contribution of individual features to the prediction. This transparency fosters clinician trust, facilitates clinical adoption, and bridges the gap between algorithmic predictions and medical decision-making. Within the multimodal risk assessment framework, *Helicobacter pylori* infection, age, and melaena emerged as the top predictors of PLGC, findings that are consistent with previous epidemiological evidence and supported by established biological mechanisms.

Compared with traditional non-invasive screening methods,^12^^,^^13^^,^^15^ the proposed model demonstrated superior specificity without compromising sensitivity, thereby reducing the risk of false positives. While existing methods often yield lower specificity and higher misdiagnosis rates, our model achieved higher AUC and sensitivity values, indicating improved diagnostic accuracy and efficiency. Furthermore, in the field of multivariate analysis combining patient symptom data with ML, this work makes a novel contribution by integrating interpretability into model development, offering a methodological framework that can be adapted to other disease screening contexts.

Recent studies have explored diverse non-invasive and AI-driven strategies for gastric cancer detection. Shah et al. emphasised in the American Gastroenterological Association Clinical Practice Update that screening and surveillance remain underutilised among individuals at high-risk despite the availability of emerging diagnostic technologies.^48^ Lopes et al. identified a saliva-derived transcriptomic signature through machine learning, demonstrating the feasibility of molecular screening.^31^ An et al. proposed a five-serum-cytokine panel for early detection of chronic atrophic gastritis,^49^ while Bu et al. used plasma extracellular vesicle metabolomics to achieve highly accurate early gastric cancer diagnosis.^50^ Zhang et al. applied hyperspectral imaging with deep learning to histopathological diagnosis of precancerous lesions,^30^ and Ma et al. introduced a tongue image–based ML model as a low-cost visual biomarker.^51^ Wang et al. developed an AI model for pre-endoscopic PLGC screening using routine clinical data,^32^ and Orăşeanu et al. systematically reviewed innovative diagnostic technologies integrating imaging and molecular analytics.^52^ Collectively, these studies highlight complementary strengths but share challenges in scalability, standardisation, and real-world deployment.

Collectively, these studies underscore that although AI- and biomarker-based approaches—spanning imaging, transcriptomics, and metabolomics—yield valuable insights; they continue to face practical constraints related to cost, generalisability, and implementation in routine care. Extending our earlier lifestyle-plus-tongue-image model,^32^ which demonstrated feasibility but required standardised image acquisition, the present study isolates the non-imaging symptom- and lifestyle-based dimension to provide a transparent, device-independent framework with SHAP-based feature attributions for clinically salient predictors (e.g., *Helicobacter pylori* infection, age, alcohol use, temperature/food preferences). In doing so, it complements image-centric pipelines by offering a clinically interpretable alternative that is well suited to resource-limited settings and can serve as an entry point in stepwise pathways (questionnaire → selective serology/imaging) for real-world deployment.

Beyond their predictive contribution, the selected features also display clear clinical and biological relevance. *Helicobacter pylori* infection, age, and family history represent classical drivers of the inflammation–atrophy–metaplasia–dysplasia cascade through chronic epithelial injury.^53^, ^54^, ^55^, ^56^ Symptom-related variables such as black stool, distending pain, acid–bile reflux, and motility dysfunction—which are hallmarks of long-standing gastritis.^57^, ^58^, ^59^ Interestingly, the PLGC-related symptoms from our analysis—vague epigastric pain, abdominal distention, preference for cold food, and bitter taste in the mouth—are largely consistent with the TCM damp-heat syndrome, in line with records in the ancient classic *Huang Di Nei Jing*, which describes chronic spleen–stomach cold–heat imbalance as an important internal condition predisposing to tumorigenesis. Moreover, lifestyle-related indicators, including alcohol intake and temperature preferences, capture behavioural exposures that influence mucosal resilience: repeated consumption of very hot foods may induce thermal injury and epithelial hyperplasia, whereas cold or processed foods can alter gastric motility and perfusion.^54^, ^55^, ^56^ Together, these interpretable, non-invasive features align well with known pathogenic mechanisms, strengthening the biological plausibility of the model and supporting its translational value for early screening of PLGC.

The developed model offers a non-invasive tool suitable for integration into primary healthcare systems, particularly in resource-limited settings. It enables rapid preliminary screening of high-risk populations, improving early case detection and guiding targeted use of confirmatory diagnostics such as endoscopy. By optimising resource allocation, the model has the potential to enhance screening coverage, reduce diagnostic delays, and ultimately improve patient outcomes at the population level.

The cost-effectiveness analysis indicates that, for comparable case detection, the model-based strategy can substantially reduce the number of endoscopies and the average cost per detected PLGC case relative to guideline-based screening, supporting scalability in resource-limited settings. However, the analysis used a simplified per-encounter framework with a single assumed unit cost and did not incorporate adverse events, downstream care, or quality-adjusted outcomes; a comprehensive health-economic evaluation with multi-centre real-world data is warranted in future work.

Several limitations should be acknowledged. First, the age structure, sex and geographic distribution of our study cohorts may limit the generalisability of the model, particularly to populations with different demographic or regional characteristics. Second, while external validation was performed, the study lacked prospective, real-world clinical trials to assess model performance in routine practice. Third, the model showed higher performance in the training dataset compared with the testing dataset, indicating a potential risk of overfitting.

In light of the substantial global burden of gastric cancer and the pressing demand for accessible early detection strategies, future research should prioritise expanding the dataset to include ethnically and geographically diverse populations from regions with high gastric cancer incidence, such as East Asia, Eastern Europe, and parts of Latin America. This would enable systematic stratified analyses to evaluate and optimise the model's applicability across demographic and regional subgroups defined by age, sex, and geography. Furthermore, future work should aim to develop a unified foundational model trained on large-scale, multi-regional data, followed by region-specific fine-tuning to further enhance the model's effectiveness and generalisability.

Integrating the model with additional non-invasive biomarkers—such as serological tests, microbiome profiles, or portable imaging—could further improve diagnostic accuracy and clinical utility. To ensure robustness, methodological refinements including regularisation techniques, more heterogeneous training data, and large-scale, independent external validations will be required. Importantly, embedding the model into prospective, multi-centre clinical trials will allow assessment of its performance in routine workflows, from primary care to tertiary hospitals.

By integrating more diverse data, combining complementary non-invasive diagnostics, and validating the model in real-world settings, future work could support scalable, non-invasive PLGC screening with broader clinical applicability in high-burden regions.

## Contributors

SL, LW, and KT conceived and designed the study. SL, PZ, LW, JL, KT, BW, JC, YL, and SD collected and analysed the data. KT, LW, JL, and YW drafted the original manuscript. SL and PZ critically reviewed and revised the manuscript for important intellectual content. All authors read and approved the final manuscript.

All authors had full access to all the data in the study and take responsibility for the integrity of the data and the accuracy of the data analysis.

## Data sharing statement

Deidentified individual participant data will be available from the corresponding author upon reasonable request and after approval of a data access proposal. It is available upon request via email: shaoli@mail.tsinghua.edu.cn.

## Declaration of interests

LW, KT, PZ, JL, BW, JC, YL, SD, YW, and SL declare that they have no competing interests.

## Appendix A Supplementary data

Supplementary Material

Translated Abstract
