# Supplementary material for: Development and validation of an interpretable machine learning model for non-invasive screening of precancerous gastric lesions using symptom and lifestyle data: a multicentre cohort study
Source: eClinicalMedicine. 2026 Jan 17;92:103756. doi: 10.1016/j.eclinm.2026.103756 (PMC12856190; doi:10.1016/j.eclinm.2026.103756)
Supplement: Supplementary Material [file mmc1.pdf]

Supplementary Material

Tables of contents

Supplementary Methods 2

Simplified cost-effectiveness analysis of guideline-based versus model-based PLGC screening 2

Supplementary Figures 3

Figure S1. Framework of the machine learning-based model for precancerous gastric lesion assessment 3

Figure S2. Feature importance for PLGC risk prediction by temperature preference using SHAP analysis 4

Figure S3 Regional characteristics of PLGC-related features. 5

Figure S4. Regional SHAP analysis of PLGC-related risk features 6

Supplementary Tables 7

Table S1. Glossary of abbreviations used in the manuscript. 7

Table S2. Participating hospitals, locations, study cohorts and ethics approvals. 10

Table S3. Community screening sites, locations and ethics information. 12

Supplementary Methods

Simplified cost-effectiveness analysis of guideline-based versus model-based PLGC screening

According to the *Chinese Guidelines for Screening, Early Detection, and Early Treatment of Gastric Cancer (2022 edition)*, individuals aged ≥ 45 years who meet any high-risk criterion (such as *H. pylori* infection, a history of chronic gastritis, a family history of gastric cancer, a high-salt diet, smoking, or heavy alcohol use) are recommended to undergo endoscopic examination. While scientifically valid, this guideline-based approach involves high cost, invasive procedures, and limited accessibility, making it difficult to implement at a population level. In contrast, our model relies solely on routinely obtainable symptom and lifestyle information, requiring no laboratory or imaging data, thus offering an extremely low marginal cost and high scalability as a pre-endoscopic screening tool.

Assuming a population size $N$with disease prevalence p, and denoting the guideline-based and model-based strategies as $G$and M, respectively, the number of individuals requiring endoscopy and the number of true positives are defined as follows:

$$E_j = N[p\,\mathrm{Se}_j + (1-p)(1-\mathrm{Sp}_j)], \mathrm{TP}_j = Np\,\mathrm{Se}_j$$

The total screening cost is

$$\mathrm{Cost}_j = E_j \times C^{\mathrm{endo}}$$

and the average cost per detected case is

$$\mathrm{CPCD}_j = \frac{\mathrm{Cost}_j}{\mathrm{TP}_j}.$$

Using the external validation set 2 (actual prevalence $p = 36.8\%$) and assuming an endoscopy cost of ¥800 per procedure, the comparison is as follows:

Strategy	Sensitivity	Specificity	AUC	Endoscopies per 10,000 (E/N×10^4)	PLGC cases detected per 10,000	CPCD (¥ per case)
Guideline	0.78	0.33	0.57	7,105	2,870	1,980
Model	0.77	0.75	0.79	4,414	2,834	1246

(Note: Parameters are based on real data from prospective external validation set and an assumed endoscopy cost of ¥800 per procedure; results are intended to illustrate comparative trends rather than serve as a formal economic evaluation.)

As shown above, compared with the guideline-based tool, the model exhibits comparable sensitivity (0.77 vs. 0.78) but higher specificity (0.75 vs. 0.33), resulting in 2,691 fewer endoscopies per 10,000 individuals screened (−37.9%; ¥2.15 million lower endoscopy expenditure. Under the same cost assumption, the average cost per detected case decreases by 37% (¥1,246 vs. ¥1,980). Furthermore, the model's higher discrimination (AUC = 0.79 vs. 0.57) supports more effective targeting of high-risk individuals. We plan to conduct a formal health-economic evaluation with multicenter real-world data in future research.

Supplementary Figures

Figure S1. Framework of the machine learning-based model for precancerous gastric lesion assessment

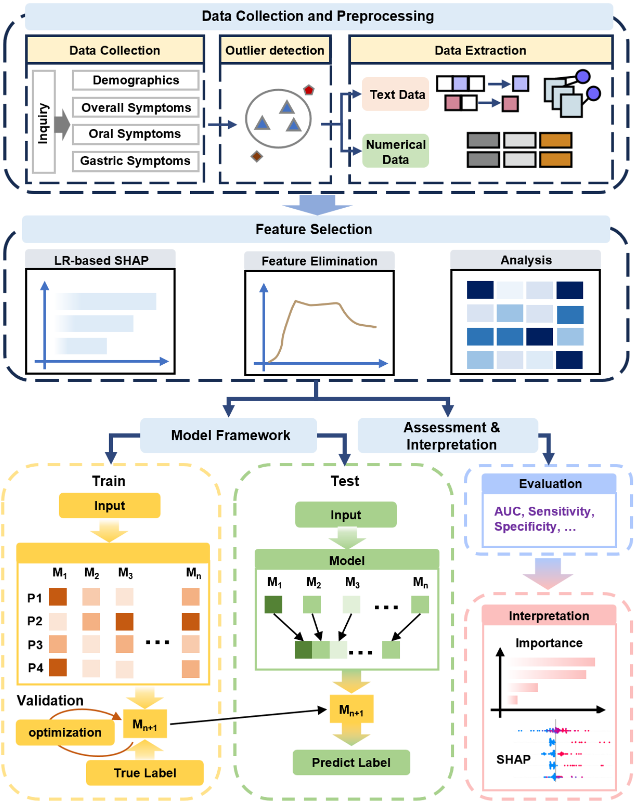

LR = Logistic Regression. SHAP = Shapley Additive Explanations. AUC = Area under the Receiver Operating Characteristic Curve.

Figure S2. Feature importance for PLGC risk prediction by temperature preference using SHAP analysis

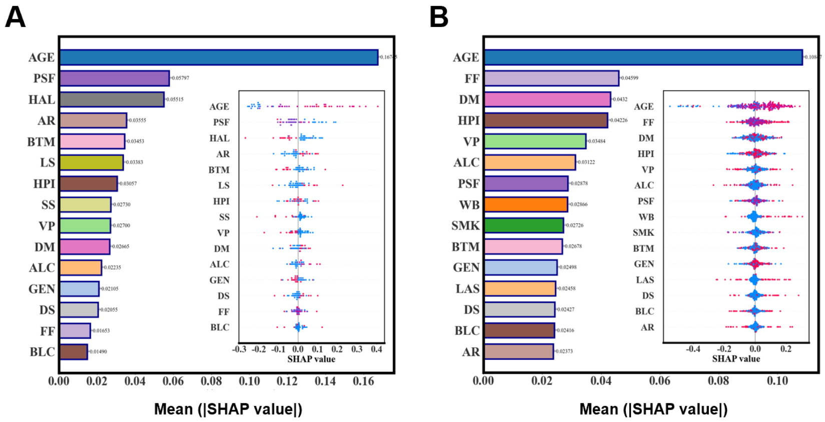

(A) Preference for cold food. (B) preference for warm food. ALC = Alcohol Consumption. AR = Acid Regurgitation. BLC = Belching. BTM = Bitter Taste in the Mouth. DM = Dry Mouth. DS = Dry Stool. FF = Fried Foods. HAL = Halitosis. HPI = Helicobacter pylori Infection. LAS = Lassitude. LS = Loose Stool. PSF = Pickled and Smoked Foods. SMK = Smoking. SS = Sticky Stool. VP = Vague Pain. WB = Water Brash. PLGC = Precancerous Gastric Lesions. SHAP = Shapley Additive Explanations.

Figure S3 Regional characteristics of PLGC-related features.

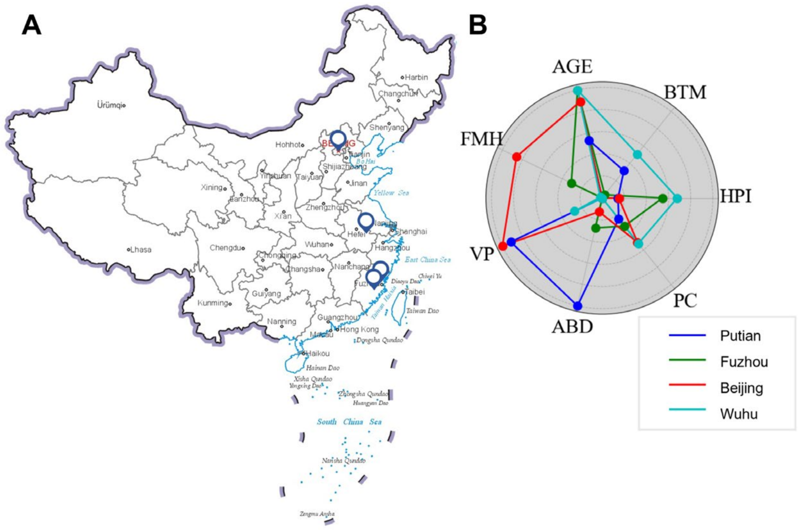

(A) Geographic distribution of participants. (B) Representative features associated with PLGC risk in four major regions, illustrated by radar charts. FMH = Family Medical History. AGE = Age. BTM = Bitter taste in the mouth. HPI = Helicobacter pylori Infection. PC = Preference for Cold Food. ABD = Abdominal Distention. VP = Vague Pain. Note: This figure was prepared using the standard base map (Map approval no. GS(2019)1651) obtained from the Standard Map Service of the Ministry of Natural Resources of the People's Republic of China. The base map has not been modified.

Figure S4. Regional SHAP analysis of PLGC-related risk features

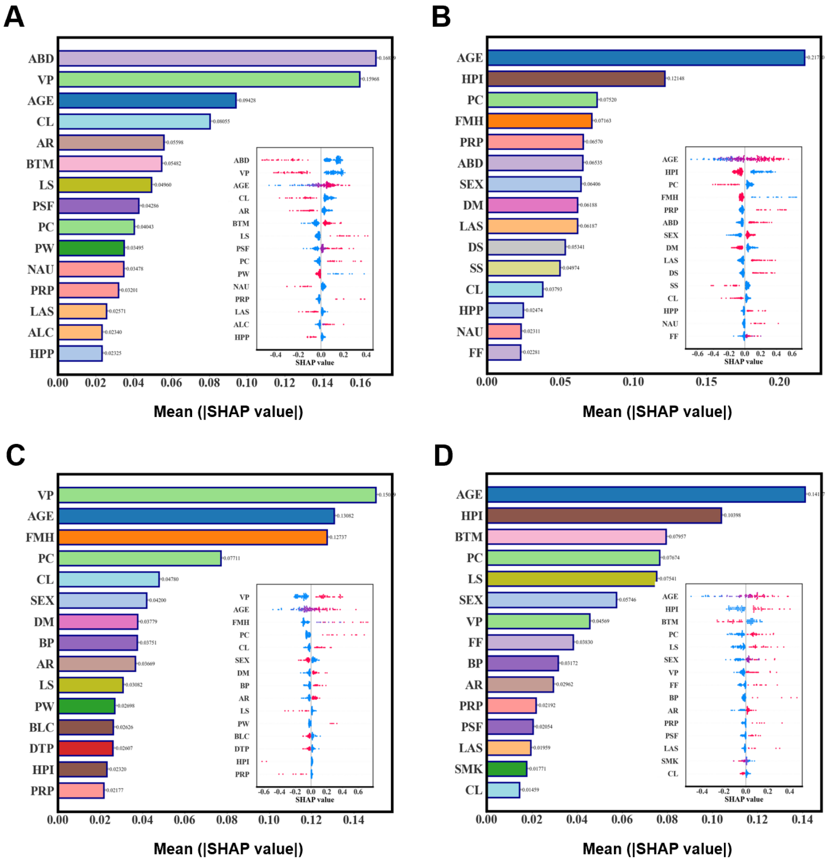

Shapley Additive Explanations plots showing the relative contribution of clinical features to PLGC risk prediction in participants from four representative cities. (A) Putian. (B) Fuzhou. (C) Beijing. (D) Wuhu. ABD = Abdominal Distention. AGE = Age. ALC = Alcohol Consumption. AR = Acid Regurgitation. BP = Burning Pain. BTM = Bitter Taste in the Mouth. CL = Cold Limbs. DM = Dry Mouth. DS = Dry Stool. DTP = Distending Pain. FF = Fried Foods. FMH = Family Medical History. GEN = GenderSex. HAL = Halitosis. HPI = Helicobacter pylori Infection. LAS = Lassitude. LS = Loose Stool. NAU = Nausea. PA = Poor Appetite. PC = Preference for Cold Food. PRP = Pricking Pain. PSF = Pickled and Smoked Foods. PW = Preference for Warm Food. SMK = Smoking. SS = Sticky Stool. VP = Vague Pain. WB = Water Brash. PLGC = Precancerous Gastric Lesions. SHAP = Shapley Additive Explanations.

Supplementary Tables

Table S1. Glossary of abbreviations used in the manuscript.

Abbreviation	Full term
ABD	Abdominal distention
ACC	Accuracy
ADB	Adaptive boosting
AGE	Age
AI	Artificial intelligence
ALC	Alcohol consumption
ANE	Anaemia
AR	Acid regurgitation
AUC	Area under the curve
AUROC	Area under the receiver operating characteristic curve
BLC	Belching
BNRIST	Beijing National Research Center for Information Science and Technology
BP	Burning pain
BS	Black stool
BTM	Bitter taste in the mouth
CL	Cold limbs
CPCD	Cost per case detected
DCA	Decision curve analysis
DM	Dry mouth
DS	Dry stool
DTP	Distending pain
EGD	Esophagogastroduodenoscopy
ELISA	Enzyme-linked immunosorbent assay
EN	British Society of Gastroenterology Guidelines for the Diagnosis of Gastric Cancer Risk
EPV	Events per variable
FF	Fried foods
FMH	Family medical history (of gastric cancer)
GBC	Gradient boosting classifier
GC	Gastric cancer
GEN	Gender
GNB	Gaussian Naïve Bayes

Abbreviation	Full term
HAL	Halitosis
HPI	Helicobacter pylori infection
HPP	Hypochondriac pain
IgG	Immunoglobulin G
KNN	K-nearest neighbours
LAS	Lassitude
LR	Logistic regression
LR_NEG	Negative likelihood ratio
LR_POS	Positive likelihood ratio
LS	Loose stool
ML	Machine learning
MOE	Ministry of Education
NAU	Nausea
NPV	Negative predictive value
PA	Poor appetite
PC	Preference for cold food
PLGC	Precancerous lesions of gastric cancer
PPV	Positive predictive value
PRP	Pricking pain
PSF	Pickled and smoked foods
PW	Preference for warm food
RF	Random forest
ROC	Receiver operating characteristic
SHAP	Shapley Additive Explanations
SMK	Smoking
SS	Sticky stool
Se	Sensitivity
Sp	Specificity
TCM	Traditional Chinese medicine
TRIPOD	Transparent Reporting of a multivariable prediction model for Individual Prognosis Or Diagnosis
TRIPOD+AI	TRIPOD extension for Artificial Intelligence–based prediction models
VP	Vague pain
WB	Water brash (clear watery regurgitation)
XGB	eXtreme Gradient Boosting

Abbreviation	Full term
ZH	Chinese Guidelines for Gastric Cancer Screening and Early Diagnosis and Treatment

Table S2. Participating hospitals, locations, study cohorts and ethics approvals.

Site type / cohort	Participating site	Address	Study role / cohort	Ethics committee	Ethics approval number(s)	Ethics model / note
Development cohort (hospital-based)	The First Affiliated Hospital of Fujian Medical University	999 Huashan Road, Changle District, Fuzhou City, Fujian Province, China	Lead hospital for development cohort	Ethics Committee of Fujian Medical University (FJMU)	FJMU-2022-120	Lead hospital within the local medical alliance; provides primary ethics approval for the development cohort.
Development cohort (hospital-based)	Putian Traditional Chinese Medicine Hospital	181 East Meiyuan Road, Licheng District, Putian City, Fujian Province, China	Alliance member hospital contributing to the development cohort	Ethics Committee of Fujian Medical University	FJMU-2022-86&12 (recognized)	Member hospital within the Fujian medical alliance; formally recognizes the ethics approval of the Fujian Medical University.
Retrospective cohort (hospital-based)	Yijishan Hospital of Wannan Medical College	2 West Zheshan Road, Jinghu District, Wuhu City, Anhui Province, China	Lead hospital for the Anhui medical alliance; contributes retrospective cases	Scientific Research and New Technology Institutional Review Board of Wannan Medical College Yijishan Hospital (WMCYH)	WMCYH-2020 (03)	Lead ethics approval for the Anhui medical alliance; covers retrospective data from alliance member hospitals.
Retrospective cohort (hospital-based)	Huangshan Traditional Chinese Medicine Hospital	59 East Huangshan Road, Tunxi District, Huangshan City, Anhui Province, China	Member hospital of the Anhui medical alliance; contributing retrospective cases	Scientific Research and New Technology Institutional Review Board of Yijishan Hospital of Wannan Medical College	WMCYH-2020 (03) (recognized)	Member hospital of the Anhui medical alliance; formally recognizes the ethics approval of Yijishan Hospital.
Retrospective cohort (hospital-based)	Ma'anshan Traditional Chinese Medicine	61 Xingfu Road, Huashan District, Ma'anshan City,	Member hospital of the Anhui medical alliance; contributing	Scientific Research and New Technology Institutional Review Board of Yijishan	WMCYH-2020 (03) (recognized)	Member hospital of the Anhui medical alliance; formally recognizes the

Site type / cohort	Participating site	Address	Study role / cohort	Ethics committee	Ethics approval number(s)	Ethics model / note
	Hospital	Anhui Province, China	retrospective cases	Hospital of Wannan Medical College		ethics approval of Yijishan Hospital.
Retrospective cohort (hospital-based)	China-Japan Friendship Hospital	2 Yinghua East Street, Chaoyang District, Beijing, China	Tertiary referral hospital contributing retrospective cases	Clinical Research Ethics Committee of China-Japan Friendship Hospital (CJFH)	CJFH-2020-10-K07; CJFH-2023-KY-174	Independent hospital IRB approval for retrospective data use.

Table S3. Community screening sites, locations and ethics information.

Screening site name	Address	Cohort	Coordinating hospital / screening team	Ethics committee	Ethics approval number
Caicuo Village Screening Site	Caicuo Village, Donghai Town, Chengxiang District, Putian City, Fujian Province, China	Prospective community-based cohort	First Affiliated Hospital of Fujian Medical University	Ethics Committee of Fujian Medical University	FJMU-2022-86&12
Shangting Village Screening Site	Shangting Village, Donghai Town, Chengxiang District, Putian City, Fujian Province, China	Prospective community-based cohort	First Affiliated Hospital of Fujian Medical University	Ethics Committee of Fujian Medical University	FJMU-2022-86&12
Bangtou Village Screening Site	Bangtou Village, Lingchuan Town, Chengxiang District, Putian City, Fujian Province, China	Prospective community-based cohort	First Affiliated Hospital of Fujian Medical University	Ethics Committee of Fujian Medical University	FJMU-2022-86&12
Kezhu Village Screening Site	Kezhu Village, Lingchuan Town, Chengxiang District, Putian City, Fujian Province, China	Prospective community-based cohort	First Affiliated Hospital of Fujian Medical University	Ethics Committee of Fujian Medical University	FJMU-2022-86&12
Shufeng Village Screening Site	Shufeng Village, Lingchuan Town, Chengxiang District, Putian City, Fujian Province, China	Prospective community-based cohort	First Affiliated Hospital of Fujian Medical University	Ethics Committee of Fujian Medical University	FJMU-2022-86&12
Xidun Village Screening Site	Xidun Village, Lingchuan Town, Chengxiang District, Putian City, Fujian Province, China	Prospective community-based cohort	First Affiliated Hospital of Fujian Medical University	Ethics Committee of Fujian Medical University	FJMU-2022-86&12
Zhangbian Village Screening Site	Zhangbian Village, Lingchuan Town, Chengxiang District, Putian City, Fujian Province, China	Prospective community-based cohort	First Affiliated Hospital of Fujian Medical University	Ethics Committee of Fujian Medical University	FJMU-2022-86&12

Screening site name	Address	Cohort	Coordinating hospital / screening team	Ethics committee	Ethics approval number
Jiangkou Community Screening Site	Jiangkou Neighborhood, Jiangkou Town, Hanjiang District, Putian City, Fujian Province, China	Prospective community-based cohort	First Affiliated Hospital of Fujian Medical University	Ethics Committee of Fujian Medical University	FJMU-2022-86&12
Xinpu Village Screening Site	Xinpu Village, Sanjiangkou Town, Hanjiang District, Putian City, Fujian Province, China	Prospective community-based cohort	First Affiliated Hospital of Fujian Medical University	Ethics Committee of Fujian Medical University	FJMU-2022-86&12
Haibin Village Screening Site	Haibin Village, Huangshi Town, Licheng District, Putian City, Fujian Province, China	Prospective community-based cohort	First Affiliated Hospital of Fujian Medical University	Ethics Committee of Fujian Medical University	FJMU-2022-86&12
Huadong Village Screening Site	Huadong Village, Huangshi Town, Licheng District, Putian City, Fujian Province, China	Prospective community-based cohort	First Affiliated Hospital of Fujian Medical University	Ethics Committee of Fujian Medical University	FJMU-2022-86&12
Huazhong Village Screening Site	Huazhong Village, Huangshi Town, Licheng District, Putian City, Fujian Province, China	Prospective community-based cohort	First Affiliated Hospital of Fujian Medical University	Ethics Committee of Fujian Medical University	FJMU-2022-86&12
Jiangdong Village Screening Site	Jiangdong Village, Huangshi Town, Licheng District, Putian City, Fujian Province, China	Prospective community-based cohort	First Affiliated Hospital of Fujian Medical University	Ethics Committee of Fujian Medical University	FJMU-2022-86&12
Yaotai Village Screening Site	Yaotai Village, Huangshi Town, Licheng District, Putian City, Fujian Province, China	Prospective community-based cohort	First Affiliated Hospital of Fujian Medical University	Ethics Committee of Fujian Medical University	FJMU-2022-86&12
Zhelang Village Screening Site	Zhelang Village, Huangshi Town, Licheng District, Putian City, Fujian Province, China	Prospective community-based cohort	First Affiliated Hospital of Fujian Medical University	Ethics Committee of Fujian Medical	FJMU-2022-86&12

Screening site name	Address	Cohort	Coordinating hospital / screening team	Ethics committee	Ethics approval number
				University	
Dapu Village Screening Site	Dapu Village, Donghai Town, Chengxiang District, Putian City, Fujian Province, China	Prospective community-based cohort	First Affiliated Hospital of Fujian Medical University	Ethics Committee of Fujian Medical University	FJMU-2022-86&12
Haitou Village Screening Site	Haitou Village, Donghai Town, Chengxiang District, Putian City, Fujian Province, China	Prospective community-based cohort	First Affiliated Hospital of Fujian Medical University	Ethics Committee of Fujian Medical University	FJMU-2022-86&12
Dongjin Village Screening Site	Dongjin Village, Lingchuan Town, Chengxiang District, Putian City, Fujian Province, China	Prospective community-based cohort	First Affiliated Hospital of Fujian Medical University	Ethics Committee of Fujian Medical University	FJMU-2022-86&12
Qiaodou Village Screening Site	Qiaodou Village, Huangshi Town, Licheng District, Putian City, Fujian Province, China	Prospective community-based cohort	First Affiliated Hospital of Fujian Medical University	Ethics Committee of Fujian Medical University	FJMU-2022-86&12
Luding Village Screening Site	Luding Village, Zhanggang Subdistrict, Changle District, Fuzhou City, Fujian Province, China	Prospective community-based cohort	First Affiliated Hospital of Fujian Medical University	Ethics Committee of Fujian Medical University	FJMU-2022-120
Langfeng Village Screening Site	Langfeng Village, Yutian Town, Changle District, Fuzhou City, Fujian Province, China	Prospective community-based cohort	First Affiliated Hospital of Fujian Medical University	Ethics Committee of Fujian Medical University	FJMU-2022-120
Sanfeng Community Screening Site	Sanfeng Neighborhood, Wuhang Subdistrict, Changle District, Fuzhou City, Fujian Province, China	Prospective community-based cohort	First Affiliated Hospital of Fujian Medical University	Ethics Committee of Fujian Medical University	FJMU-2022-120
Hujing Village	Hujing Village, Wenwusha Town, Changle	Prospective community-	First Affiliated Hospital of	Ethics Committee	FJMU-2022-

Screening site name	Address	Cohort	Coordinating hospital / screening team	Ethics committee	Ethics approval number
Screening Site	District, Fuzhou City, Fujian Province, China	based cohort	Fujian Medical University	of Fujian Medical University	120
Wenling Village Screening Site	Wenling Village, Wenling Town, Changle District, Fuzhou City, Fujian Province, China	Prospective community-based cohort	First Affiliated Hospital of Fujian Medical University	Ethics Committee of Fujian Medical University	FJMU-2022-120
Houdong Village Screening Site	Houdong Village, Tantou Town, Changle District, Fuzhou City, Fujian Province, China	Prospective community-based cohort	First Affiliated Hospital of Fujian Medical University	Ethics Committee of Fujian Medical University	FJMU-2022-120
Shouzhi Village Screening Site	Shouzhi Village, Songxia Town, Changle District, Fuzhou City, Fujian Province, China	Prospective community-based cohort	First Affiliated Hospital of Fujian Medical University	Ethics Committee of Fujian Medical University	FJMU-2022-120
Jinfeng Community Screening Site	Jinfeng Neighborhood, Jinfeng Town, Changle District, Fuzhou City, Fujian Province, China	Prospective community-based cohort	First Affiliated Hospital of Fujian Medical University	Ethics Committee of Fujian Medical University	FJMU-2022-120
Sanxi Village Screening Site	Sanxi Village, Jiangtian Town, Changle District, Fuzhou City, Fujian Province, China	Prospective community-based cohort	First Affiliated Hospital of Fujian Medical University	Ethics Committee of Fujian Medical University	FJMU-2022-120
Minsha Village Screening Site	Minsha Village, Hunan Town, Changle District, Fuzhou City, Fujian Province, China	Prospective community-based cohort	First Affiliated Hospital of Fujian Medical University	Ethics Committee of Fujian Medical University	FJMU-2022-120
Xianjie Village Screening Site	Xianjie Village, Heshang Town, Changle District, Fuzhou City, Fujian Province, China	Prospective community-based cohort	First Affiliated Hospital of Fujian Medical University	Ethics Committee of Fujian Medical University	FJMU-2022-120

Screening site name	Address	Cohort	Coordinating hospital / screening team	Ethics committee	Ethics approval number
Hanghui Community Screening Site	Hanghui Neighborhood, Hangcheng Subdistrict, Changle District, Fuzhou City, Fujian Province, China	Prospective community-based cohort	First Affiliated Hospital of Fujian Medical University	Ethics Committee of Fujian Medical University	FJMU-2022-120
Yangxia Village Screening Site	Yangxia Village, Guhuai Town, Changle District, Fuzhou City, Fujian Province, China	Prospective community-based cohort	First Affiliated Hospital of Fujian Medical University	Ethics Committee of Fujian Medical University	FJMU-2022-120
