# Supplementary material for: Development and validation of an interpretable machine learning model for non-invasive screening of precancerous gastric lesions using symptom and lifestyle data: a multicentre cohort study
Source: eClinicalMedicine. 2026 Jan 17;92:103756. doi: 10.1016/j.eclinm.2026.103756 (PMC12856190; doi:10.1016/j.eclinm.2026.103756)
Supplement: Translated Abstract [file mmc2.docx]

***The following translations in Chinese were submitted by the authors and we reproduce them as supplied. They have not been peer reviewed. Our editorial processes have only been applied to the original abstract in English, which should serve as reference for this manuscript.***

**Chinese Abstract**

**背景** 胃癌前病变（precancerous gastric lesions，PLGC）是胃癌发生发展过程中的关键阶段，在该阶段及时干预可显著降低死亡率。然而，目前的筛查策略主要依赖内镜检查，这种方式具有侵入性、成本高且在资源有限地区往往难以获得。我们旨在基于症状和生活方式数据，开发并验证一种用于无创 PLGC 筛查的可解释机器学习（Machine Learning，ML）模型。

**方法** 在这项多中心研究中，我们纳入了正在接受或计划接受上消化道内镜检查、且无既往恶性肿瘤诊断的符合条件的成年人。开发队列包括1,034名受试者，来自两家医院，入组时间为2022年11月16日至2023年4月7日。基于该队列的症状和生活方式数据构建开发数据集，并随机划分为训练集（n = 620）、内部验证集（n = 207）和保留测试集（hold-out test set，n = 207）。外部性能评估基于两个独立队列：一是来自其它四家医院的回顾性医院队列（n = 630，2018年 5月21日至2023年7月30日），二是来自32个社区筛查点的前瞻性社区队列（n = 847，2023年6月21日至2023 年11月7日）。我们构建了一个堆叠集成模型，通过整合七个基础学习器（Gaussian Naïve Bayes、Logistic Regression、K-Nearest Neighbours、Gradient Boosting Classifier、Extreme Gradient Boosting、Random Forest 和Adaptive Boosting）来预测参与者是否存在 PLGC，并采用 Shapley Additive Explanations（SHAP）实现临床可解释性。模型性能与两种基于指南的筛查策略进行比较：一是《中国胃癌筛查及早诊早治指南》，二是《英国胃肠病学会胃癌风险指南》。评价指标包括受试者工作特征曲线下面积（AUC; 95% CI）、灵敏度、特异度、阳性预测值和阴性预测值。

**结果** 共纳入2511名受试者（男性871例，占34.7%；女性1,640例，占65.3%）。在开发队列中，1034名受试者中有509例（49.2%）存在PLGC；在回顾性验证队列中，630名受试者中有331例（52.5%）存在PLGC；在前瞻性验证队列中，847名受试者中有312例（36.8%）存在PLGC。该模型在无创PLGC筛查中表现稳健：内部保留测试集的AUC为0.82（95% CI 0.77–0.87），外部回顾性验证集的AUC为0.80（95% CI 0.78–0.82），前瞻性验证集的AUC为0.79（95% CI 0.77–0.81）。在所有数据集中，模型的AUC均优于两种基于指南的筛查策略，提升幅度为0.18–0.35（内部保留测试集：0.82 (95% CI 0.77-0.87) vs. 0.47 (95% CI 0.42-0.53) / 0.48 (95% CI 0.42-0.53)；外部回顾性验证集：0.80 (95% CI 0.78-0.82) vs. 0.62 (95% CI 0.60-0.64) / 0.58 (95% CI 0.55-0.60)；前瞻性验证集：0.79 (95% CI 0.77-0.81) vs. 0.57 (95% CI 0.54-0.59) / 0.52 (95% CI 0.50-0.55)；均 p < 0.001）。在成本效果分析中，这一优势转化为相较基于指南的工具，每检出一例PLGC的平均成本降低37.1%。SHAP 分析进一步识别出15个关键预测因子，其中包括幽门螺杆菌感染、年龄和黑便等。

**解读** 一种整合症状和生活方式信息的可解释机器学习模型，在医院人群和社区人群中进行的PLGC无创筛查中，其表现均优于基于指南的筛查策略。然而，由于队列的年龄结构和地区分布，该模型的推广性可能受到一定限制；未来研究应纳入更多无创指标以进一步优化筛查模型，并在更广泛的外部人群中进行验证，同时推动真实世界中的实施与评估。

**资助** 国家自然科学基金；国家中医药管理局创新团队与人才培养项目；教育部学科攻关试点项目。

**关键词** 胃癌癌前病变；机器学习；筛查工具；症状与生活方式。
